# Responses of sequential and hierarchical phenological events to warming and cooling in alpine meadows

**DOI:** 10.1038/ncomms12489

**Published:** 2016-08-18

**Authors:** Xine Li, Lili Jiang, Fandong Meng, Shiping Wang, Haishan Niu, Amy M. Iler, Jichuan Duan, Zhenhua Zhang, Caiyun Luo, Shujuan Cui, Lirong Zhang, Yaoming Li, Qi Wang, Yang Zhou, Xiaoying Bao, Tsechoe Dorji, Yingnian Li, Josep Peñuelas, Mingyuan Du, Xinquan Zhao, Liang Zhao, Guojie Wang

**Affiliations:** 1 Key Laboratory of Alpine Ecology and Biodiversity, Institute of Tibetan Plateau Research, Chinese Academy of Sciences, Beijing 100101, China; 2 Key Laboratory of Adaptation and Evolution of Plateau Biota, Northwest Institute of Plateau Biology, Chinese Academy of Sciences, Xining 810008, China; 3 CAS Center for Excellence in Tibetan Plateau Earth Science of the Chinese Academy of Sciences, Beijing 100101, China; 4 Naqu Integrated Observation and Research Station of Ecology and Environment, Tibet University and Institute of Tibetan Plateau Research of the Chinese Academy of Sciences, Lasa 850012, China; 5 University of Chinese Academy of Sciences, Beijing 100049, China; 6 Aarhus Institute of Advanced Studies, Aarhus University, Høegh-Guldbergs Gade 6B, Aarhus CDK-8000, Denmark; 7 Binhai Research Institute in Tianjin, Tianjin 300457, China; 8 CREAF, Cerdanyola del Vallès, Barcelona, Catalonia 08193, Spain; 9 CSIC, Global Ecology Unit CREAF-CEAB-CSIC-UAB, Cerdanyola del Vallès, Barcelona, Catalonia 08193, Spain; 10 Institute for Agro-Environmental Sciences, National Agriculture and Food Research Organization, Tsukuba 305-8604, Japan; 11 Oregon State University Agriculture and Natural Resource Program at Eastern Oregon University, La Grande, Oregon 97850, USA

## Abstract

Organisms' life cycles consist of hierarchical stages, from a single phenological stage (for example, flowering within a season), to vegetative and reproductive phases, to the total lifespan of the individual. Yet phenological events are typically studied in isolation, limiting our understanding of life history responses to climate change. Here, we reciprocally transfer plant communities along an elevation gradient to investigate plastic changes in the duration of sequential phenological events for six alpine species. We show that prolonged flowering leads to longer reproductive phases and activity periods when plants are moved to warmer locations. In contrast, shorter post-fruiting leaf and flowering stages led to shorter vegetative and reproductive phases, respectively, which resulted in shorter activity periods when plants were moved to cooler conditions. Therefore, phenological responses to warming and cooling do not simply mirror one another in the opposite direction, and low temperature may limit reproductive allocation in the alpine region.

## Abstract (web summary)

Describing changes in the timing of life history events is critical to understanding effects of climate change. Wang *et al*. relocated plant communities up and down elevation gradients and found that warming lengthened the reproductive and activity phases, while cooling reduced the vegetative and reproductive phases.

## Graphical abstract

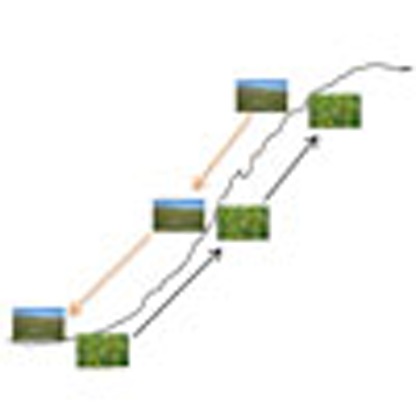

Isolated phenological events (for example, green-up, flowering) have largely shaped our understanding of phenology as a bioindicator of climate change^1–4^; yet in reality, phenological events are inter-related and hierarchical. For example, flower bud formation, flowering and fruit/seed maturation compose the reproductive phase of angiosperms; furthermore, vegetative and reproductive phases compose the annual total aboveground activity period of plants in seasonal habitats (hereafter activity period; Fig. 1). Certain phenological stages within the plant life cycle may limit later stages^5^,^6^ and therefore affect the responsiveness of other phenological stages to environmental change. Thus, assessing an entire sequence of phenological events can reveal which individual stages have the largest effect on composite phenologies, and provide comprehensive insight into life history responses to climate change^6^,^7^,^8^,^9^.

The degree to which plants respond to temperature changes by adjusting vegetative and reproductive phases should depend in part on the effect of temperature on resource allocation to growth versus reproduction. In addition, temperature-dependent physiological processes can regulate the duration of plant life history events. Plant growth and reproduction depend on the same internal resource pool^10^,^11^, and plants allocate a small percentage of their total resources to sexual reproduction in arctic and alpine regions^12^, presumably because they live in harsh environments with a short time window to complete their life cycles. Yet climate warming is resulting in a longer period of time that is conducive to growth and reproduction in many seasonal habitats^13^,^14^,^15^,^16^. Conversely, warmer environments may impose new constraints on the duration of phenological events if faster rates of development occur under warmer temperatures^8^,^17^. Given the harsh abiotic environment in alpine habitats, we expect that warmer temperatures will reduce constraints on allocation to sexual reproduction, in which case we expect longer reproductive phases and activity periods. Following this same logic, we expect reproductive phases to be shortened more so than vegetative phases under cooling, for a total reduction in activity periods.

In this study we reciprocally transferred intact plant communities in 1 m by 1 m blocks of soil at 3,200, 3,400, 3,600 and 3,800 m above sea level in the Qilian Mountains of China. Our experiment provides insight into how plants may adjust sequences of hierarchical phenological events within the life cycle (Fig. 1) to warmer temperatures expected under future climate change in addition to temperature anomalies, such as short-term cooling events^18^,^19^. In addition, by examining plastic responses in phenological duration to both warmer and cooler temperatures, our study improves our understanding of the basic role of temperature in shaping the relationships among phenological events in the plant life cycle, a need that has been highlighted by the widespread effects of climate change on phenological events^20^. Intact soil blocks were transferred across all elevations in 2007. The phenology of six common perennial plants was monitored from 2008 to 2010 at all sites. These plants included two early-flowering sedge species, *Kobresia humilis* (Kh) and *Carex scabrirostris* (Cs); two mid-summer flowering forbs *Potentilla anserine* (Pa) and *P. nivea* (Pn); and two mid-summer flowering grasses, *Poa pratensis* (Pp) and *Stipa aliena* (Sa). We measured the durations of six phenological stages in tagged individual plants of each species: leaf-out, flower bud, flowering, fruiting, post-fruiting leaf and leaf colouring^21^ (Fig. 1) with 3–4 day intervals at each elevation. We report a metric of phenological sensitivity to temperature changes from the reciprocal transplants for each phenological event: change in duration divided by absolute change in temperature between each donor and receptor site (**Δ**days °C^−1^).

Here we find that warming significantly prolongs the duration of flowering, which is primarily responsible for longer reproductive phases and activity periods, but cooling significantly reduces both reproductive and vegetative phases and activity periods. Our results show that the temperature sensitivities of flowering duration to warming and post-fruiting leaf duration to cooling are greater compared with other phenological stages. Thus, the responses of phenological stages to warming and cooling do not simply mirror one another in the opposite direction, and low temperature may limit the reproductive allocation in the alpine region.

## Results

### Temperature sensitivity of hierarchical phenological events

Inter-related phenological events within each hierarchy were analysed using a repeated-measures multivariate analysis of variance (MANOVA). The sensitivities of the activity period and vegetative and reproductive phases to temperature were significantly affected by temperature change direction (that is, warming and cooling) and/or species and their interaction (Tables 1 and 2). Warming prolonged the activity period and the reproductive phase, while cooling shortened the activity period and both vegetative and reproductive phases for all species (Fig. 2a–c). Warming significantly prolonged the activity period by an average of 15.7 days °C^−1^ across species (Fig. 2c), and the reproductive phase accounted for on average 83% of the increase in the duration of the activity period under warming (range 69–99% across species; Fig. 2d). In contrast, cooling shortened the activity period by an average of 14.8 days °C^−1^ across species (Fig. 2c), and the reproductive phase accounted for on average 44.8% of the decrease (range 20–68% across species) in the duration of the activity period under cooling (Fig. 2d).

### Temperature sensitivity of individual phenological event

Plant species, temperature change direction and their interaction all significantly affected the sensitivity of duration to temperature in the six individual phenological stages (Table 3). Warming significantly prolonged the duration of the reproductive phase (11.1–15.1 days °C^−1^ across species) (Fig. 2b), which was mainly caused by the prolonged duration of flowering for all plant species (which ranged from 7.4 to 11.3 days °C^−1^) (Fig. 3a–f). Longer fruiting also contributed to longer reproduction in four of the six species (0–5.7 days °C^−1^) (Fig. 3c–f) and longer flower budding in four of six species (Fig. 3a,c,e,f), but these stages always contributed less than the flowering stage. Warming also significantly prolonged the duration of the vegetative phase in three species (1.7–5.5 days °C^−1^) (Fig. 2a), which primarily derived from the prolonged duration of leaf colouring (1.5–4.4 days °C^−1^) (Fig. 3a,c–f; Cs is the exception (Fig. 3b)). In contrast, cooling significantly shortened the duration of the vegetative phase (4.6–9.6 days °C^−1^) (Fig. 2a), which resulted primarily from the shortening of the post-fruiting leaf stage for all species (5.1–10.1 days °C^−1^) (Fig. 3a–f). A shortened duration of fruit-set contributed to shorter reproductive phases (2.2–6.0 days °C^−1^) under cooling for all plant species (Fig. 3a–f), although for Pa and Pn, shorter flowering stages contributed more than fruiting (Fig. 3c,d).

### Correlations among phenological events

There were significant negative correlations between the sensitivity of the vegetative (VP) and reproductive phases (RP) to warming and cooling regardless of whether the analysis was pooled across all species (Table 4) or conducted separately for each species (Supplementary Tables 2–13; except for Pp (Supplementary Table 11) and Sa (Supplementary Table 13) under cooling). In other words, high sensitivities of RP to temperature correspond to low sensitivities of VP to temperature and vice versa. Significant negative correlations were found between some individual stages within VP, and between them and individual stages within RP under warming and cooling (Table 1), which may be indicative of tradeoffs among sequential events. However, positive correlations among individual reproductive stages shows that high sensitivities in flowering are correlated with high sensitivities in fruiting and flower budding when pooled across all species (Table 1). The exceptions to this pattern are significant negative correlations between sensitivities of fruit bud and flower durations for Kh under warming (Supplementary Table 2) and for Pa under both warming and cooling (Supplementary Tables 6 and 7), and between sensitivities of flower and fruit durations for Pa (Supplementary Tables 6 and 7) and Pn under cooling (Supplementary Table 9), respectively.

## Discussion

Our reciprocal transfer experiment revealed substantial plasticity in the duration of phenological events in six alpine plant species and allowed us to identify the individual stages that drive changes in composite phenophases. In all three years of the study, moving plants down in elevation to warmer temperatures prolonged activity periods of individuals in all six species, mainly because of longer reproductive phases; in particular flowering. Conversely, moving plants up in elevation to cooler temperatures shortened both vegetative and reproductive phases, for shorter annual activity periods in individuals of all six species. These patterns are consistent despite the inclusion of three different plant life forms in our study: sedges, grasses and forbs, which is suggestive of a common response to the alpine environment. Unlike other experimental studies in high-elevation or high-latitude sites, we found no evidence of lag or build-up effects of warming on plant phenology^14^,^22^. Instead, sensitivity of duration to temperature was consistent across years in all species, despite interannual variation in temperature (for example, 2008 was cooler than 2009 and 2010 by 0.6–0.7 °C on average)^3^.

Most studies understandably focus on the effects of warming on plant phenophases in response to climate change, but in our study we also address a need for more basic research on relationships among inter-related phenological events^23^ by asking whether responses to cooling are simply the opposite of responses to warming or whether events respond differentially. The outcome depends on hierarchy level, which highlights the utility of considering phenological sequences within a hierarchical framework. For the upper hierarchies of the activity period, reproductive phase and vegetative phase, it appears that opposite responses are indeed observed under warming versus cooling (longer versus shorter). However, cooler temperatures do not simply have the opposite effect of warmer temperatures on the duration of individual life history events, especially within the vegetative phase. Therefore, it should not be assumed that effects of cooler temperatures on the duration of phenological events will simply mirror responses to warmer temperatures in the opposite direction.

The onset of reproduction signals a change in resource allocation from growth and survival to fecundity within the plant life cycle^24^,^25^. Longer reproductive phases under warming and shorter phases under cooling suggest that allocation to reproduction is probably increased and reduced according to temperature in these alpine plants. Our results are consistent with the observation that alpine plants allocate a small proportion of their resources to sexual reproduction under harsh environmental conditions^12^. Thus we find no evidence of a faster rate of development of reproductive structures under warmer temperatures as seen in other studies^8^,^17^. Instead, our results support the hypothesis that reproductive allocation in these plants is generally constrained by cold-temperature conditions in the alpine environment.

Longer reproduction and activity periods under warmer temperatures were primarily driven by longer flowering stages in all species (Fig. 2d). The flowering stage accounted for 70% of the extension to the reproductive phase under warming and for 58% of the extension to the activity period on average across species. Advanced first flowering appears to be the main contribution to prolonged flowering duration under warming, as opposed to later last flowering^4^. Some experiments have found either longer flowering or maintenance of flowering duration under warmer temperatures, depending on the species^17^,^26^,^27^,^28^, whereas other experiments have found a shorter duration of flowering and other individual reproductive stages under warming^6^,^22^,^29^. This variation across species in how the duration of flowering responds to warming may in part reflect different warming methods (open-top chambers versus infrared heaters versus transplants) in addition to ecosystem type—specifically the timing of abiotic extremes that may constrain individual stages within a growing season. For example, divergence in entire reproductive phases and flowering stages at the species level has been detected in two habitats with extreme abiotic conditions in the middle of the growing season—high temperatures and drought—so that a mid-season gap in reproduction appears at the community level^29^,^30^. In sum, plastic responses to climate change are likely to include phenological shifts away from parts of the season where abiotic conditions are becoming more extreme and toward parts of the season where abiotic extremes are being attenuated.

The response of flowering to warming may be attributed to genetic and physiological mechanisms, and the genetic basis of flowering is the most well understood plant life history phase^31^,^32^. The onset of flowering occurs earlier under warming and later under cooling in these six species^3^, which agrees with laboratory research on genetic pathways regulating how the timing of flowering responds to temperature in *Arabadopsis thaliana*^33^,^34^,^35^,^36^,^37^. Thus, the responses of flowering duration in this study are probably mediated through similar pathways at the genetic level that determine the switch to allocation to reproduction. However, in contrast to what we find here, a recent gene regulatory model predicts shorter flowering durations under climate warming in *Anemone halleri*, a perennial relative of *A. thaliana*^38^. We suspect this discrepancy is related to effects of warmer temperatures on vernalization requirements^35^,^36^,^38^, which appear not to play a role over the range of temperature variation in our study but could be important under continued climatic warming.

The population and community-level consequences of extended phenological stages under climate change will depend on biotic interactions with antagonists and mutualists, in addition to which type of interaction has a stronger effect on plant fitness. Longer flowering durations in individual plants may reduce the likelihood of phenological mismatch with animal pollinators^39^,^40^,^41^,^42^,^43^ and may also compensate or buffer against potentially declining pollinator populations under climate change^44^,^45^. Longer flowering durations of individuals may also alter patterns of assortative mating, with potential consequences for additive genetic variation within the population, depending on whether synchrony among individuals is maintained or increased^46^,^47^. Longer activity periods could increase exposure of plants to herbivory and disease in both vegetative and floral tissue. Indeed, over longer time scales that span multiple plant generations, biotic interactions like the ones described above are likely to drive adaptive responses in phenological duration^48^, which could differ from the short-term plastic responses reported in our study.

In contrast to the individual reproductive stages that largely changed in concert with one another under warming and cooling, different individual vegetative stages accounted for the effects of warming and cooling on the duration of the vegetative phase (Table 1). In general, leaf senescence was extended under warming, whereas the post-fruit leaf stage was shortened under cooling (Fig. 3). Shorter post-fruit leaf stages are consistent with a strategy of maintaining allocation to growth and reproduction under cooling, instead of allocating to tissue maintenance following reproduction, although in two species the fruiting or flowering stages were reduced in a similar magnitude to the post-fruit leaf stage. Leaf senescence is an integrated response of leaf cells to endogenous developmental and external environmental conditions^49^, and our results are consistent with another study that found longer leaf coloration periods in deciduous trees under warmer fall temperatures^50^. Longer growing seasons often lead to increased primary production and carbon sequestration^13^,^51^,^52^. However, longer activity periods may not translate into increased carbon sequestration in our study because senescence was the main driver of longer vegetative phases and longer reproduction was the main driver of longer activity periods under warming. Furthermore, the leaf-out stage was only longer under warming in one species. The balance between respiration and photosynthesis under elevated temperatures will ultimately determine whether longer activity periods result in increased or decreased primary production^53^.

Temperature is not the only factor that varied over the elevation gradient in our study and is only one of several factors that may affect plant phenology under climate change^54^. Although higher elevation sites tended to be drier, the 3,600 m site had the highest soil moisture. Thus, our conclusions of the effects of temperature are unlikely to be confounded by concurrent changes in soil moisture because these two abiotic factors do not precisely covary across our four sites. Although photoperiod can play a role in the regulation of alpine plant life history events^55^, photoperiod does not vary across our sites that are separated by a maximum distance of 9 km. Increasing atmospheric levels of CO_2_ can also extend the duration of life history events under warming, and will most likely affect vegetative stages directly^28^. Additionally, the timing of spring snowmelt often predicts the timing of flowering in high-elevation and high-latitude environments^2^,^26^, but in our study area the ground is usually snow-free unless there is a large snow event^56^; thus, the timing of snowmelt is unlikely to be an important phenological cue in our study. Our experimental protocol controlled for plant community context and below-ground soil communities, but other biotic factors may vary across the sites. For example, the activity and effectiveness of animal pollinators may have varied across the sites, but pollinators would only affect the reproduction of the two forb species, and tradeoffs between pollinator visitation rates and pollinator effectiveness across elevation gradients can compensate for such variation^57^,^58^. Finally, we focused on the duration of events at the individual plant-level in this study, but the hierarchical nature of phenological events can be categorized in various ways. For example, measuring the duration of individual leaves and flowers would provide further insight into the consequences of phenological responses to temperature change^23^,^59^.

A main goal of ecological research is to understand what drives the abundance and distribution of species in space and time, and climate change presents ecologists with a unique opportunity to advance basic research in temporal ecology. Rapidly shifting species' phenologies under climate change have brought to light that we still need an improved understanding of how various abiotic and biotic factors affect the duration of life cycle events in order to predict species' responses to climate change. Extended phenological durations may or may not be adaptive under climate change, but in our study species, the duration of flowering, post-fruiting leaf, and senescence should be particularly sensitive to climate change in the near future.

## Methods

### Experimental site and data collection

The experimental site and design are described elsewhere in detail^3^,^21^. In brief, the experiment was conducted at Haibei Alpine Meadow Ecosystem Research Station of the Chinese Academy of Sciences, located at latitude 37°37′N, and longitude 101°12′E along a 3,200–3,800 m elevational gradient (3,200, 3,400, 3,600 and 3,800 m) on the south slope of the Qilian Mountains in Qinghai, China. The four sites included four different plant communities within 9 km of one another (maximum distance between lowest and highest elevation). Although the dominant plant species vary across these four communities, the six study species are commonly found at all four sites (for more details see^3^,^21^). At the centre of each site, HOBO weather stations (Onset Computer Corporation, Cape Cod, Massachusetts, USA) were used to monitor soil temperature at 5 cm depth and soil moisture at 20 cm depth. Data were sampled at 1 min intervals, and 30 min averages were stored in the data logger. Annual average soil temperatures at 5 cm depth were 3.9, 2.5, 2.0 and 0.4 °C, and annual average soil moistures at 20 cm depth were 11.8, 11.3, 12.7 and 10.2% at 3,200, 3,400, 3,600 and 3,800 m during the experimental period, respectively. Photoperiod is consistent across the four sites because of their close proximity^3^,^21^.

Twelve intact soil blocks (100 × 100 cm wide × 30–40 cm deep) (30 cm depth at 3,800 m due to shallower soil layer) with attached vegetation from each elevation were reciprocally transferred across the elevation gradient after the soils started to thaw in early May, 2007 (12 soil blocks per elevation × 4 elevations, for a total of 48 soil blocks or plots). There were three replicate transfers from each elevation, and these intact soil blocks were fully randomized throughout the study site. Three blocks from each elevation were removed and then reinstated at the same site to produce experimental control blocks that had been handled as similarly as possible to those blocks moved to other elevations. Thus, there were six levels of warming (transferred down) and six levels of cooling (transferred up).

### Measurement of phenological events

**Figure 1:**
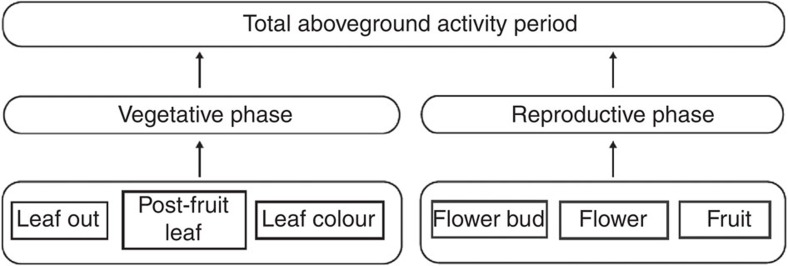
Diagram of hierarchical phenological events measured in this study. Each phenological event was measured as the number days from start to end, or the duration of the event. The vegetative phase is the sum of the duration of individual-level vegetative events that occur before and after the reproductive phase. The activity period refers to the total length of the aboveground activity period.

Six common perennial plant species from these blocks that are common at each of the four sites were chosen for monitoring of multiple phenological events at each elevation during the growing seasons of 2008 to 2010 (refs 3, 21). The plant species observed were two early-spring flowering sedge species (*K. humilis* (Kh) and *C. scabrirostris* (Cs)), which flower before May, and four mid-summer flowering species (two grasses: *Poa pratensis* (Pp) and *S. aliena* (Sa) and two forbs: *P. anserine* (Pa) and *P. nivea* (Pn)), which flower between late June and July. Ten individuals for forbs and 10 stems for graminoids for each plant species in each plot were marked during autumn 2007, so that individual plants could be followed throughout the growing season. The same individuals were monitored from 1 year to the next. Observations were made at an interval of 3 or 4 days from early April to the end of October in each year, and recordings were made of the onset and end dates of six phenological events, listed here in chronological order: leaf emergence, flower budding, flowering, fruit-set for forbs or seed-set for graminoids, post-fruit/seeding vegetative stage and leaf-colouring (number of days from first yellowing or browning of leaves to complete discoloration). Because phenology was assessed at 3–4 day intervals, onset and end dates of each phenological event were estimated; for onset, we used the average of the first date on which the event was recorded and the date of the prior census in which the event was not recorded (vice versa for end date). For example, if flowering was observed on 5 June for the first time, and the census before that on 1 June had no flowering, the estimated onset date was 3 June. The duration of each phenological event was the number of days between the estimated starting and ending date of the event for each individual plant or stem. We calculated a plot-level average duration across the 10 individuals or stems for each phenological event and species. Note that unlike the reproductive stage, the vegetative phase is not continuous and is the sum of the duration of each of the three vegetative stages in Fig. 1. Leaf colouring represents the switch from growth to dormancy, so we classify it as a vegetative stage that determines the end of the activity period (consistent with ref. 56). The reproductive phase excluded the event of seed ripeness because this was difficult to monitor in the field. The activity period represents the number of days from the earliest date on which a leaf was observed to the latest date on which a leaf retained colour. No data were obtained at 3,600 m in 2010 because mice destroyed the plots.

### Data analysis

The response variable in our analysis is a measure of the sensitivity of phenological duration to temperature changes from the reciprocal transplants: change in duration per change in °C (**Δ**days °C^−1^). For example, if the difference in mean flowering duration between a receptor and donor site is 5 days, this is divided by the temperature difference between the two sites, say 5 °C, for a sensitivity of 1 day °C^−1^. Each species-level response is an average across 10 individuals per plot, and this calculation was performed for each phenological event for each species in every plot.

Phenological responses to warming and cooling within each hierarchy level were analysed with the method repeated-measures MANOVA (Tables 2 and 3), except total activity period, which was analysed with a repeated-measures ANOVA (Table 1) because it is a single event. MANOVA investigates whether there are statistically significant mean differences among groups in a linear combination of several dependent variables^60^. Several phenological responses within each hierarchy level are correlated with one another (Table 4 and Supplementary Tables 2–13), indicating that a MANOVA is necessary to test for an overall effect of predictors on the multiple phenological responses. Pillai's statistic (the default) was applied to the MANOVA output. Only minor differences in results were observed with the use of other statistics (Wilks and Hotelling–Lawley). Repeated-measures ANOVA was used to estimate the contributions of the factors year, species and temperature change direction (two levels, that is, warming and cooling) to the sensitivity of the duration of the activity period to temperature (Table 1). The base package of R-3.2.3 (ref. 61) was used to conduct statistical analyses.

**Figure 2:**
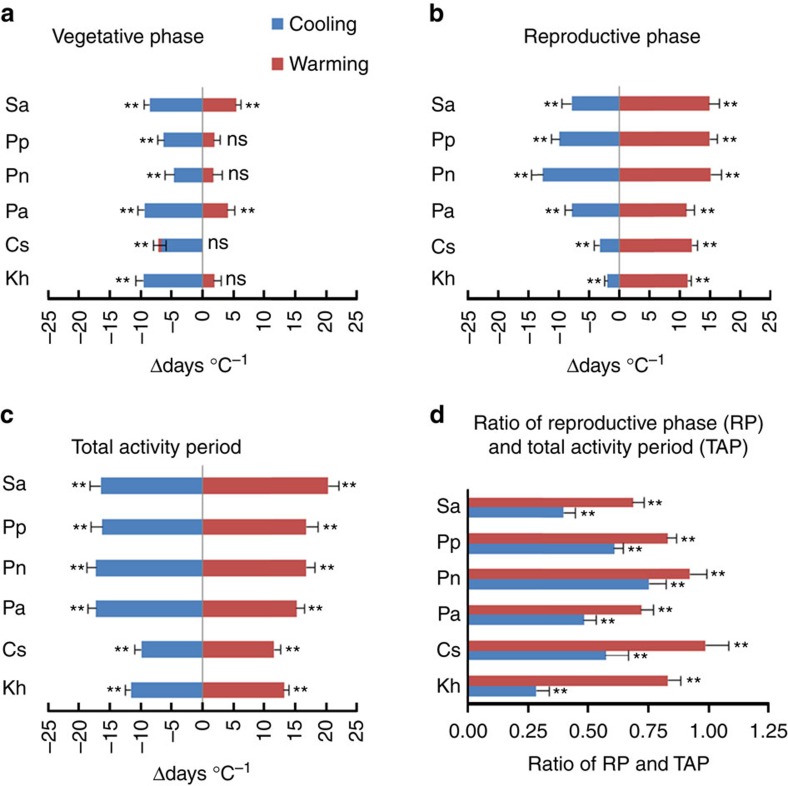
Temperature sensitivity of duration change of phenological events. The panels include the vegetative phase (**a**), reproductive phase (**b**), activity period (**c**) and their ratios of reproduction to activity period (**d**) under warming and cooling conditions. A *t*-test was used for each phenological event and species, and the *α* was adjusted following a Bonferroni correction. The mean value of phenological sensitivity in each plot in each year was the unit of replication (*n*=45 for each *t*-test under warming and cooling conditions, respectively). Kh, *K. humilis*; Cs, *C. scabrirostris*; Pa, *P. anserine*; Pn, *P. nivea*; Pp, *Poa pratensis*; and Sa, *S. aliena*. Δdays was the difference between receptor site (that is, away site) and donor site (that is, home site). Positive values indicate a prolonged phenological stage and negative values a shortened phenological stage when transferred compared with donor site. Means±s.e. are shown in the figure. ‘*', ‘**' in the figure are the significant difference at 0.004 and 0.0008 levels between receptor site and donor site. All *P* values were <0.0001 except vegetative phase for Kh, Cs, Pn and Pp under warming, which were 0.031, 0.727, 0.002 and 0.101, respectively.

**Figure 3:**
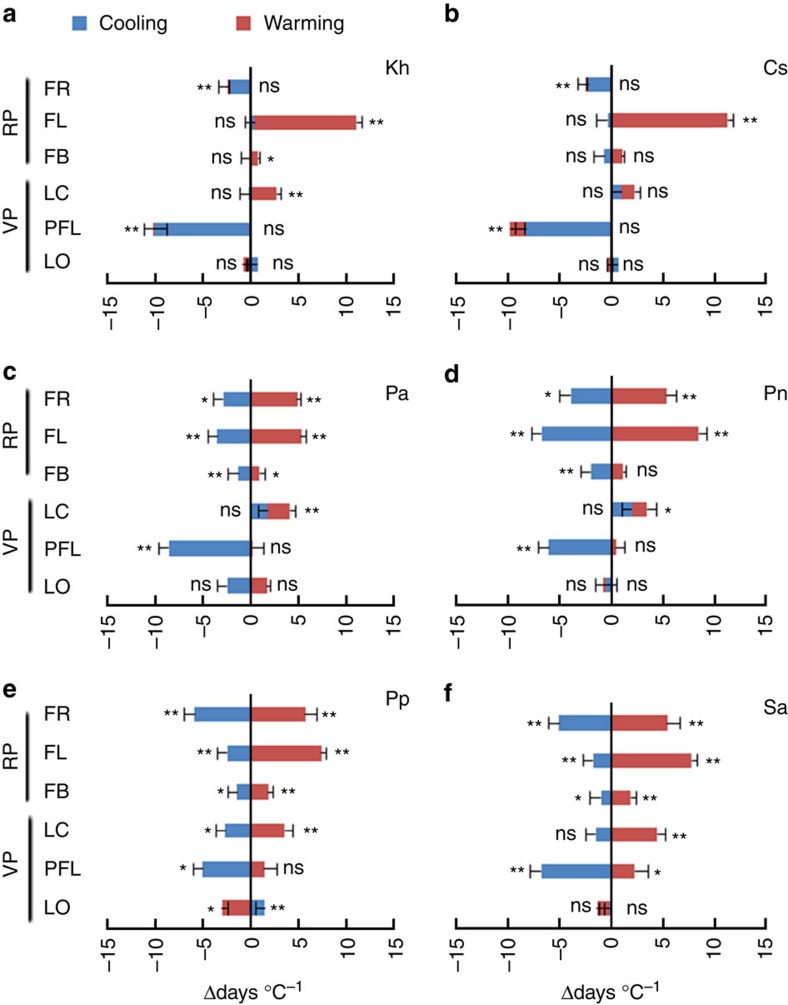
Temperature sensitivity of duration change of individual phenological stages. The panels include six phenological stages of each species under warming and cooling (Δdays °C^−1^). VP, vegetative phase; RP, reproductive phase; LO, leafing-out; PFL, post-fruiting leaf; LC, leaf colouring; FB, fruit bud; FL, flowering; FR, fruit-set/seeding. Δdays was the difference between receptor site (that is, away site) and donor site (that is, homesite). Positive and negative values were prolonged and shortened phenological duration when transferred compared with donor site, respectively. Kh, *K. humilis* (**a**); Cs, *C. scabrirostris* (**b**); Pa, *P. anserine* (**c**); Pn, *P. nivea* (**d**); Pp, *Poa pratensis* (**e**); and Sa, *S. aliena* (**f**). A *t*-test was used for each phenological event and species, and the *α* was adjusted following a Bonferroni correction. Since 12 tests were presented in figures, the *α* level for each individual test was set to 0.05/12. Significant differences were reported at *P*<0.004 and 0.0008 levels in the text. The mean value of phenological sensitivity in each plot in each year was the unit of replication (*n*=45 for each *t*-test under warming and cooling, respectively). Means±s.e. are shown in **a**–**f**. ‘*' and ‘**' are significant difference at 0.004 and 0.0008 levels between receptor site and donor site. ns means no significant difference at 0.004 level. All *P* values were shown in Supplementary Table 1.

The effects of cooling and warming on sensitivity of duration for different phenological events were analysed by comparison to the controls (the plots transferred within the same elevations). A *t*-test was used for each phenological event and species, and the *α* was adjusted following a Bonferroni correction (Figs 2 and 3). Since 12 tests were presented both in Figs 2 and 3, the *α* level for each individual test was set to 0.05/12. Significant differences were reported at *P*<0.004 and 0.0008 levels in the text. The mean value of phenological sensitivity in each plot in each year was the unit of replication (*n*=45 for each *t*-test under warming and cooling, respectively).

Simple correlation analysis was performed among the sensitivities of duration change to temperature for all phenological events to determine their relationships in direction and magnitude for each species and across all species during the experimental periods. Since 36 tests were presented in Table 1, the significance level for each individual test was set to 1/36, a conservative correction for multiple comparisons (that is, significant correlations were reported at *P*<0.0014 and 0.0003 levels in the text).

### Data availability

The data that support the findings of this study are available from the corresponding author upon request.

## Additional information

**How to cite this article:** Li, X. E. *et al*. Responses of sequential and hierarchical phenological events to warming and cooling in alpine meadows. *Nat. Commun.* 7:12489 doi: 10.1038/ncomms12489 (2016).

## Supplementary Material

Supplementary Information Supplementary Tables 1-13

## Figures and Tables

**Table 1:** Temperature sensitivity of duration change for total aboveground activity period.

	**Df**	**Sum Sq**	**Mean Sq**	***F*** **value**	***Pr*****(>*****F***)
Year	1	134	134	0.861	0.354
Species (S)	5	1,309	262	1.677	0.138
TCD	1	325,116	325,116	2,083.094	<0.001
S * TCD	5	7,163	1,433	9.179	<0.001

ANOVA, analysis of variance; TCD, temperature change direction (that is, warming and cooling).

Repeated-measures ANOVA is used to test the effects of year, species and temperature change direction on sensitivity of duration change to temperature for the total aboveground activity period.

**Table 2:** Temperature sensitivity of duration change for vegetative and reproductive phases.

	**df**	**Pillai**	**approx** ***F***	**num df**	**den df**	***Pr*****(>*****F***)
Year	1	0.00248	0.65	2	526	0.521
Species (S)	5	0.04017	2.16	10	1054	0.0181
TCD	1	0.81048	1,124.71	2	526	<0.001
S * TCD	5	0.17802	10.30	10	1054	<0.001

MANOVA, multivariate analysis of variance; TCD, temperature change direction (that is, warming versus cooling).

Repeated-measures MANOVA analysis of sensitivity of duration to temperature for both vegetative and reproductive phases is used as the multivariate response and species, year and TCD as predictors.

**Table 3:** Temperature sensitivity of duration change for six individual phenological stages.

	**df**	**Pillai**	**approx** ***F***	**num df**	**den df**	***Pr*****(>*****F***)
Year	1	0.01675	1.48	6	522	0.1821
Species (S)	5	0.33889	6.37	30	2,630	<0.001
TCD	1	0.82313	404.89	6	522	<0.001
S * TCD	5	0.53236	10.45	30	2,630	<0.001

MANOVA, multivariate analysis of variance; TCD, temperature change direction (that is, warming versus cooling).

Repeated-measures MANOVA analysis of sensitivity of duration to temperature for the six individual phenological stages is used as the multivariate response and species, year and TCD as predictors.

**Table 4:** Correlations among temperature sensitivities of phenological duration.

	**Leaf-out**	**Flower bud**	**Flower**	**Fruit**	**Post-fruit leaf**	**Leaf colour**	**VP**	**RP**
*Warming*								
Flower bud	−0.12							
Flower	−0.60**	0.28**						
Fruit	−0.14	0.55**	0.03					
Post-fruit leaf	−0.41**	−0.25**	−0.08	−0.10				
Leaf colour	−0.18	−0.07	−0.04	0.05	0.05			
VP	0.37**	−0.31**	−0.55**	−0.14	0.37**	0.63**		
RP	−0.48**	0.72**	0.70**	0.70**	−0.17	−0.01	−0.50**	
AP	−0.31**	0.62**	0.44**	0.71**	0.05	0.40**	0.09	0.82**
								
*Cooling*								
Flower bud	0.18							
Flower	−0.38**	0.15						
Fruit	0.06	0.28**	0.19*					
Post-fruit leaf	−0.31**	−0.26**	0.13	0.04				
Leaf colour	−0.29**	−0.20	−0.11	−0.47**	−0.29**			
VP	0.04	−0.33**	−0.16	−0.26**	0.73**	0.21*		
RP	−0.10	0.54**	0.66**	0.81**	0.01	−0.41**	−0.34**	
AP	−0.06	0.25**	0.49**	0.56**	0.65**	−0.21*	0.47**	0.67**

AP, activity period; RP, reproductive phase; VP, vegetative phase.

* and ** indicate significant correlations at *P*<0.0014 and 0.0003 levels, respectively.

The temperature sensitivity of each phenological event is included under warming and cooling, across all three years of the study (2008–2010). All species were pooled for ease of interpretation, because results are largely consistent if each species is analysed separately (Supplementary Tables 1–12). The sample unit is the mean sensitivity value for each species in each plot and year (*n*=270 for warming and for cooling, respectively).
