# Supplementary material for: Responses of sequential and hierarchical phenological events to warming and cooling in alpine meadows
Source: Nat Commun. 2016 Aug 18;7:12489. doi: 10.1038/ncomms12489 (PMC4992149; doi:10.1038/ncomms12489)
Supplement: Supplementary Information — Supplementary Tables 1-13 [file ncomms12489-s1.pdf]

Supplementary Tables

Supplementary Table 1 p-values in figure 3 of the main text using t-test (n = 45)

		LO	PFL	LC	FB	FL	FR
Kh	Cooling	0.063	<0.000	0.740	0.835	0.493	<0.000
	Warming	0.107	0.833	<0.000	0.001	<0.000	0.577
Cs	Cooling	0.107	<0.000	0.145	0.199	0.473	<0.000
	Warming	0.456	0.065	0.261	0.059	<0.000	0.339
Pa	Cooling	0.056	<0.000	0.063	<0.000	<0.000	0.005
	Warming	0.177	0.953	<0.000	0.004	<0.000	<0.000
Pn	Cooling	0.460	<0.000	0.021	0.001	<0.000	0.003
	Warming	0.726	0.465	0.001	0.016	<0.000	<0.000
Pp	Cooling	0.002	0.001	0.001	<0.000	<0.000	<0.000
	Warming	<0.000	0.025	<0.000	<0.000	<0.000	<0.000
Sa	Cooling	0.549	<0.000	0.152	0.002	<0.000	<0.000
	Warming	0.097	0.001	<0.000	<0.000	<0.000	<0.000

LO: leafing-out; PFL: post-fruiting leaf; LC: leaf coloring; FB: fruit bud; FL: flowering; FR: fruit-set/seeding. Kh: *Kobresia humilis*; Cs: *Carex scabrirostris*; Pa: *Potentilla anserine*; Pn: *P. nivea*; Pp: *Poa pratensis*; Sa: *Stipa aliena*.

Supplementary Table 2 Correlations among the temperature sensitivities of duration changes of different phenological events under warming for *Kobresia humilis* (n = 45)

	Leaf out	Flower bud	Flower	Fruit	Post-fruit leaf	Leaf coloring	Vegetative phase	Reproductive phase
Flower but	-0.331*							
Flower bud	-0.759**	0.373*						
Fruit	0.016	0.267	0.007					
Post-fruit leaf	-0.271	0.099	0.098	0.526**				
Leaf coloring	0.420**	0.017	-0.180	-0.579**	-0.745**			
Vegetative phase	0.834**	-0.152	-0.554**	-0.301	-0.469**	0.824**		
Reproductive phase	-0.721**	0.563**	0.947**	0.292	0.229	-0.295	-0.586**	
Activity period	-0.229	0.573**	0.728**	0.121	-0.089	0.288	0.069	0.768**

* and ** indicate significant correlations at $p < 0.0014$ and 0.0003 levels, respectively.

Supplementary Table 3 Correlations among the temperature sensitivities of duration changes of different phenological events under cooling for *Kobresia humilis* (n = 45)

	Leaf out	Flower bud	Flower	Fruit	Post-fruit leaf	Leaf coloring	Vegetative phase	Reproductive phase
Flower but	0.350*							
Flower bud	-0.553**	-0.306						
Fruit	0.183	0.061	-0.256					
Post-fruit leaf	-0.377*	-0.251	-.378*	-0.341*				
Leaf coloring	0.416**	0.331*	0.086	0.208	-0.525**			
Vegetative phase	0.032	-0.056	-.589**	-0.265	0.862**	-0.092		
Reproductive phase	-0.366*	0.075	.819**	0.228	-0.653**	0.312	-0.768**	
Activity period	-0.167	-0.033	-.336*	-0.232	0.806**	0.053	0.929**	-0.477**

* and ** indicate significant correlations at p < 0.0014 and 0.0003 levels, respectively.

Supplementary Table 4 Correlations among the temperature sensitivities of duration changes of different phenological events under warming for *Carex scabrirostris* (n = 45)

	Leaf out	Flower bud	Flower	Fruit	Post-fruit leaf	Leaf color	Vegetative phase	Reproductive phase
flower bud	-0.269							
flower	-0.085	0.782**						
fruit	0.469**	0.163	0.446**					
post-fruit leaf	-0.225	-0.236	-0.523**	-0.319				
leaf color	0.137	-0.704**	-0.414**	-0.258	-0.174			
Vegetative phase	0.246	-0.826**	-0.699**	-0.294	0.395**	0.800**		
Reproductive phase	-0.053	0.852**	0.978**	0.525**	-0.473**	-0.536**	-0.767**	
Activity period	0.207	0.355*	0.702**	0.471**	-0.273	0.106	-0.018	0.655**

* and ** indicate significant correlations at p < 0.0014 and 0.0003 levels, respectively.

Supplementary Table 5 Correlations among the temperature sensitivities of duration changes of different phenological events under cooling for *Carex scabrirostris* (n = 45)

	Leaf out	Flower bud	Flower	Fruit	Post-fruit leaf	Leaf color	Vegetative phase	Reproductive phase
Flower bud	-0.258							
Flower	-0.218	0.458**						
Fruit	0.630**	-0.216	0.073					
Post-fruit leaf	-0.222	0.369*	-0.326*	-0.340*				
Leaf color	0.053	-0.702**	-0.514**	-0.010	-0.399**			
Vegetative phase	0.114	-0.064	-0.714**	-0.172	0.809**	0.128		
Reproductive phase	-0.031	0.749**	0.838**	0.291	-0.091	-0.691**	-0.491**	
Activity period	0.100	0.546**	-0.096	0.050	0.815**	-0.429**	0.696**	0.284

* and ** indicate significant correlations at p < 0.0014 and 0.0003 levels, respectively.

Supplementary Table 6 Correlations among the temperature sensitivities of duration changes of different phenological events under warming for *Potentilla anserine* (n = 45)

	Leaf out	Flower bud	Flower	Fruit	Post-fruit leaf	Leaf color	Vegetative phase	Reproductive phase
Flower bud	0.638**							
Flower	-0.854**	-0.618**						
Fruit	0.190	0.714**	-0.478**					
Post-fruit leaf	-0.796**	-0.789**	0.584**	-0.296				
Leaf color	-0.367*	-0.056	0.597**	-0.120	-0.114			
Vegetative phase	0.807**	0.349*	-0.544**	-0.062	-0.583**	0.012		
Reproductive phase	-0.497**	0.295*	0.388**	0.611**	0.099	0.461**	-0.494**	
Activity period	-0.099	0.544**	0.128	0.667**	-0.227	0.538**	0.016	0.861**

* and ** indicate significant correlations at $p < 0.0014$ and 0.0003 levels, respectively.

Supplementary Table 7 Correlations among the temperature sensitivities of duration changes of different phenological events under cooling for *Potentilla anserine* (n = 45)

	Leaf out	Flower bud	Flower	Fruit	Post-fruit leaf	Leaf color	Vegetative phase	Reproductive phase
Flower bud	0.641**							
Flower	-0.865**	-0.560**						
Fruit	0.161	0.402**	-0.144					
Post-fruit leaf	-0.444**	-0.169	0.715**	0.032				
Leaf color	-0.540**	-0.601**	0.499**	-0.821**	0.174			
Vegetative phase	0.376*	0.121	-0.046	-0.460**	0.393**	0.326*		
Reproductive phase	-0.308*	0.184	0.434**	0.810**	0.477**	-0.469**	-0.375*	
Activity period	0.028	0.275	0.370*	0.373*	0.779**	-0.165	0.491**	0.623**

* and ** indicate significant correlations at $p < 0.0014$ and 0.0003 levels, respectively.

Supplementary Table 8 Correlations among the temperature sensitivities of duration changes of different phenological events under warming for *Potentilla nivea* (n = 45)

	Leaf out	Flower bud	Flower	Fruit	Post-fruit leaf	Leaf color	Vegetative phase	Reproductive phase
Flower bud	0.527**							
Flower	-0.490**	0.084						
Fruit	0.322*	0.673**	-0.297					
Post-fruit leaf	-0.703**	-0.840**	-0.045	-0.459**				
Leaf color	-0.059	0.371*	0.349*	0.095	-0.245			
Vegetative phase	0.330*	-0.106	-0.405**	-0.101	0.187	0.416**		
Reproductive phase	0.164	0.846**	0.283	0.812**	-0.619**	0.350*	-0.303*	
Activity period	0.286	0.851**	0.157	0.817**	-0.585**	0.511**	0.026	0.945**

* and ** indicate significant correlations at $p < 0.0014$ and 0.0003 levels, respectively.

Supplementary Table 9 Correlations among the temperature sensitivities of duration changes of different phenological events under cooling for *Potentilla nivea* (n = 45)

	Leaf out	Flower bud	Flower	Fruit	Post-fruit leaf	Leaf color	Vegetative phase	Reproductive phase
Flower bud	0.564**							
Flower	-0.077	0.416**						
Fruit	0.402**	0.612**	0.018					
Post-fruit leaf	-0.852**	-0.643**	0.187	-0.557**				
Leaf color	-0.183	-0.551**	0.064	-0.630**	0.367*			
Vegetative phase	-0.434**	-0.662**	0.178	-0.704**	0.753**	0.846**		
Reproductive phase	0.423**	0.851**	0.446**	0.879**	-0.519**	-0.580**	-0.629**	
Activity period	0.101	0.414**	0.739**	0.408**	0.095	0.111	0.202	0.634**

* and ** indicate significant correlations at $p < 0.0014$ and 0.0003 levels, respectively.

Supplementary Table 10 Correlations among the temperature sensitivities of duration changes of different phenological events under warming for *Poa pratensis* (n = 45)

	Leaf out	Flower bud	Flower	Fruit	Post-fruit leaf	Leaf color	Vegetative phase	Reproductive phase
Flower bud	-0.819**							
Flower	-0.721**	0.407**						
Fruit	-0.857**	0.677**	0.705**					
Post-fruit leaf	-0.066	0.065	-0.167	0.147				
Leaf color	-0.267	0.146	-0.004	0.364*	0.397**			
Vegetative phase	0.497**	-0.438**	-0.577**	-0.307*	0.703**	0.553**		
Reproductive phase	-0.902**	0.700**	0.906**	0.917**	-0.020	0.170	-0.528**	
Activity period	-0.734**	0.535**	0.687**	0.878**	0.438**	0.563**	0.036	0.830**

* and ** indicate significant correlations at $p < 0.0014$ and 0.0003 levels, respectively.

Supplementary Table 11 Correlations among the temperature sensitivities of duration changes of different phenological events under cooling for *Poa pratensis* (n = 45)

	Leaf out	Flower bud	Flower	Fruit	Post-fruit leaf	Leaf color	Vegetative phase	Reproductive phase
Flower bud	-0.687**							
Flower	-0.427**	0.085						
Fruit	-0.600**	0.085	0.653**					
Post-fruit leaf	-0.103	-0.468**	0.572**	0.640**				
Leaf color	-0.363*	0.631**	-0.336*	-0.201	-0.737**			
Vegetative phase	0.029	-0.548**	0.414**	0.541**	0.909**	-0.521**		
Reproductive phase	-0.711**	0.342*	0.872**	0.887**	0.512**	-0.116	0.360*	
Activity period	-0.478**	-0.045	0.816**	0.893**	0.822**	-0.348*	0.763**	0.878**

* and ** indicate significant correlations at $p < 0.0014$ and 0.0003 levels, respectively.

Supplementary Table 12 Correlations among the temperature sensitivities of duration changes of different phenological events under warming for *Stipa aliena* (n = 45)

	Leaf out	Flower bud	Flower	Fruit	Post-fruit leaf	Leaf color	Vegetative phase	Reproductive phase
Flower bud	-0.727**							
Flower	-0.774**	0.599**						
Fruit	-0.790**	0.697**	0.913**					
Post-fruit leaf	-0.093	0.113	-0.109	-0.217				
Leaf color	-0.651**	0.482**	0.254	0.138	0.490**			
Vegetative phase	0.140	-0.069	-0.387**	-0.542**	0.877**	0.560**		
Reproductive phase	-0.830**	0.777**	0.947**	0.981**	-0.127	0.261	-0.429**	
Activity period	-0.845**	0.823**	0.845**	0.801**	0.320*	0.582**	0.049	0.881**

* and ** indicate significant correlations at $p < 0.0014$ and 0.0003 levels, respectively.

Supplementary Table 13 Correlations among the temperature sensitivities of duration changes of different phenological events under cooling for *Stipa aliena* (n = 45)

	Leaf out	Flower bud	Flower	Fruit	Post-fruit leaf	Leaf color	Vegetative phase	Reproductive phase
Flower bud	-0.390**							
Flower	-0.269	-0.074						
Fruit	-0.410**	0.064	0.806**					
Post-fruit leaf	-0.304	-0.463**	0.613**	0.728**				
Leaf color	-0.206	0.312*	-0.570**	-0.712**	-0.613**			
Vegetative phase	-0.234	-0.474**	0.076	0.007	0.571**	0.227		
Reproductive phase	-0.457**	0.233	0.853**	0.973**	0.611**	-0.618**	-0.072	
Activity period	-0.529**	-0.025	0.810**	0.883**	0.836**	-0.446**	0.432**	0.869**

* and ** indicate significant correlations at $p < 0.0014$ and 0.0003 levels, respectively.
